# Supplementary material for: MAUDE: inferring expression changes in sorting-based CRISPR screens
Source: Genome Biol. 2020 Jun 3;21:134. doi: 10.1186/s13059-020-02046-8 (PMC7268349; doi:10.1186/s13059-020-02046-8)
Supplement: Supplementary file 1 — Additional file 1. Review history. [file 13059_2020_2046_MOESM1_ESM.docx]

Review History

**First round of review**

**Reviewer 1**

**Were you able to assess all statistics in the manuscript, including the appropriateness of statistical tests used?**

No

**Were you able to directly test the methods?**

No

**Comments to author:**

In this manuscript the authors have developed the authors have developed MAUDE (Mean Alterations Using Discrete Expression) for pooled, sorting based CRISPR expression screen. MAUDE quantifies guide-level effects by modeling the distribution of cells across sorting expression bins. The authors show that MAUDE significantly improves over previous approaches (edgeR, DESeq2). MAUDE is implemented as an R package available on GitHub.
Overall this is a significant and novel method to improve the analysis of pooled, sorting based CRISPR screens. Nevertheless, several key questions need to be addressed in the revision.

Major points:
- It's unclear whether MAUDE can visualize individual sgRNA ranking and enrichment/depletion? This feature is useful for protein-coding gene-targeted sgRNA libraries to prioritize sgRNAs for validation and to rule out off-target effects.
- Can MAUDE be used for non-sorting CRISPR screens (eg, cell viability-based screens)? If so, a comparison with additional analysis tool (such as MAGeCK by Shirley Liu lab) is necessary.
Minor point:
- A table summarizing the pros and cons of MAUDE vs standard CRISPR screen algorithm (both sorting-based and non-sorting-based screens) will help lay audience appreciate the value of this new method.

**Reviewer 2**

**Were you able to assess all statistics in the manuscript, including the appropriateness of statistical tests used?**

Yes

**Were you able to directly test the methods?**

No

**Comments to author:**

MAUDE: Inferring expression changes in sorting-based CRISPR screens

De Boer and coauthors present MAUDE (Mean Alterations Using Discrete Expression), a computational method for analyzing pooled CRISPR screens with FACS to quantify the impact of each of thousands of gRNA on a target gene's expression. The data type is novel, and there is a lack of and a need for principled and sound guidelines for how to best analyze these data. Candidate options include (i) competent assembly of known statistical building blocks, (ii) repurposing more specialized tools that are known to work for other data types such as RNA-seq differential expression, and (iii) entirely new statistical methodology.

The ms in its current form seems to claims to be (iii). In fact, it is closer to (i), yet fails to take into account statistical "basics", and ends up reinventing the wheel, which turns out to be a bit eccentric, as explained below.

Specific points:

1.) The main claim to novelty of this ms is the statistical method, outlined on page 4 line 54 (4:54) until 6:8 and 13:32-13:49. However, the procedure looks similar to mixture modeling and the EM-algorithm (e.g., Raftery's work, https://www.stat.washington.edu/raftery/Research/mbc.html and https://stat.washington.edu/mclust), but with less mathematical sophistication and more heuristics. It would be important to put the current work into such context and highlight what is new or different.

Even more fundamentally, the basic problem that the method is trying to solve is to test for a difference between two empirical distributions, and it is not clear that any of the attempted sophistication is actually needed. There is a plethora of statistical tests (and literature, experience) for this basic question, such as t-test, Wilcoxon test or KS-test. In the authors' comparison, only the heavy tanks edgeR, DESeq2 and a so-called "original" log-ratio method are considered. If the proposed method turns out to be better than these basic statistical tests, it would be helpful to get some interpretation of "why?".

2.) The description of the experimental designs and the data that this method is intended for could be more explicit. It is implicit in the manuscript, but readers would benefit from a clearer delineation, perhaps in lieu of the list of assumptions at the beginning of the Results section.

Indeed, this list of assumptions is troubling since it does not indicate which of these assumptions is sufficient or necessary, and no formal proof is given. From a practical point of view, users would be either interested in necessary assumptions, i.e., assumptions which if not met imply that the method is definitely invalid, or verifiable sufficient assumptions, i.e., assumptions that can be demonstrated to hold. The assumptions that are actually stated are, however, neither of this. They are at the same time too strong (i.e., the method is likely to essentially function even if they do not hold) and unverifiable. I believe, however, that this might be primarily a problem of presentation, and think that this part of the ms could be best fixed by (i) replacing "assumptions" by a description of the experimental designs and the properties of the data that the method is designed for; and (ii) adopting an "ends justify the means" approach, i.e., to show the validity of the method by the validity/quality of its outputs, rather than of its inputs.

3.) The Stouffer method for combining z-scores is unrobust, e.g., in a situation with 99 small z-scores and 1 that is very large, their sum (Stouffer method) can still be large, and thus "significant"; and this seems unsatisfactory.

4.) Assumption "the perturbations do not differentially affect cell viability": I understand that this is partly because you use the unsorted input library as a reference. Would it be possible to determine the guide distribution in the cell pool just before sorting for use as a reference? The timepoint before cell sorting should in any case be included as a control, even if it is only to show that the bassumption is met. Can the authors illustrate this assumption in an experimental dataset?

5.) Assumption "expression is distributed approximately (log) normally". Please show (real) data where this is the case. Both normal and log-normal distributions are implausible (indeed impossible) for count based data, which are non-negative and discrete. A more commonly used and apparently useful model for count data are the Gamma-Poisson or Negative Binomial distributions, why are these not used here?

6.) "we require [...] that the screen includes non-targeting control guides that should not affect expression." In the simulations, the authors use 1000 targeting guides and 1000 non-targeting control gRNAs. This ratio of 1:1 is far away from a typical screening library used in this type of screens (in the CD69 publication by Simeonov et al., ~10,900 targeting gRNAs vs. 2,244 controls). I suggest changing the targeting:control ratio in the simulations to make it more realistic.

7.) In their analysis of CD69 regulation, the authors state that their method is able to detect new regulatory elements. However, this example only concerns one gene, there is no experimental validation, and the statistics in Fig. 3A-D are also only calculated on this one example. It would be more convincing if the authors could showcase the benefits of MAUDE in multiple independent datasets.

8.) I highly appreciate the fact the authors provide an R package via their GitHub page. The documentation (manual pages, examples, vignette, unit tests) seem minimalistic, and to really become a re-usable tool, that should in my opinion be extended.

Minor comment:

Line 45: missing word / typo: Elements can defined a priori,

**Authors Response**

**Point-by-point responses to the reviewers’ comments:**

*We thank the Reviewers for their thorough review of our manuscript, which we have significantly improved following their comments and suggestions. In addition to addressing their concerns, we have updated the manuscript with additional feedback we received on the concomitant bioRxiv preprint.*

*Here, we summarize the main reviewer points, which we address individually below.*

*We have explained the needs MAUDE addresses in the field of CRISPR screens, and how it differs from existing methods (R1 and R2).*

*We expand our analysis to recent CRISPRi and CRISPRa screens (that we recently had accepted for publication (Ray et al. 2020. Nature Comms. (available online March 6th))) to showcase MAUDE’s performance on additional experimental datasets.*

*We have added an additional method for comparison to MAUDE (i.e., log fold change of the high bin vs. unsorted cells; response to bioRxiv comment).*

*We have clarified several questions*

*We show how to inspect individual guide effects, both in the manuscript and in the online GitHub tutorials (R1 and R2).*

*We have clarified the nature of data generated by a CRISPR cell-sorting experiment (R2).*

*Rephrasing of “non-targeting” to “negative control” guides to distinguish cases where the appropriate control guides are targeting (bioRxiv).*

*Clarified the meaning of a MAUDE guide Z score, and its relationship with % expression (bioRxiv)*

**Response to Reviewer #1:**

In this manuscript, the authors have developed the authors have developed MAUDE (Mean Alterations Using Discrete Expression) for pooled, sorting based CRISPR expression screen. MAUDE quantifies guide-level effects by modeling the distribution of cells across sorting expression bins. The authors show that MAUDE significantly improves over previous approaches (edgeR, DESeq2). MAUDE is implemented as an R package available on GitHub.

Overall this is a significant and novel method to improve the analysis of pooled, sorting based CRISPR screens. Nevertheless, several key questions need to be addressed in the revision.

*We thank the Reviewer for their appreciation of our approach!*

Major points:

- It's unclear whether MAUDE can visualize individual sgRNA ranking and enrichment/depletion? This feature is useful for protein-coding gene-targeted sgRNA libraries to prioritize sgRNAs for validation and to rule out off-target effects.

*We thank the Reviewer for this point (also related to Reviewer #2’s 3rd point p. 7 below on Stouffer Z scores). This desirable feature is incorporated into MAUDE. The individual Z-scores for each guide are retained in the MAUDE output, and can be inspected. While we focused on aggregating individual guide effects (to reduce noise, especially in non-coding element screens), the Reviewer is correct that this is an important feature, in particular for ORF-targeting screens. We clarified this in the text (p. 6). We have also added examples to the online tutorial illustrating how to inspect individual guides.*

- Can MAUDE be used for non-sorting CRISPR screens (eg, cell viability-based screens)? If so, a comparison with additional analysis tool (such as MAGeCK by Shirley Liu lab) is necessary.

*We thank the Reviewer for their comment. MAGeCK and MAUDE are complementary but not overlapping. We do not think MAUDE could be applied to the selection-type screens for which MAGeCK is useful. Conversely, the assumptions made by MAGeCK make it inappropriate for the sorting-type screens for which we designed MAUDE. The “standard” analysis tools applied to sorting-based CRISPR screens previously were not intended to analyze these data, or were rudimentary. The advantages of MAUDE are that it is designed to analyze these kinds of data, and MAUDE does a better job in their analysis. We have clarified the intended niche fulfilled by MAUDE in the text (p. 3).*

Minor point:

- A table summarizing the pros and cons of MAUDE vs standard CRISPR screen algorithm (both sorting-based and non-sorting-based screens) will help lay audience appreciate the value of this new method.

*As note above MAUDE is interested for different purposes than MAGeCK, and we clarify this in the revised manuscript (p. 3).*

**Response to Reviewer 2**

De Boer and coauthors present MAUDE (Mean Alterations Using Discrete Expression), a computational method for analyzing pooled CRISPR screens with FACS to quantify the impact of each of thousands of gRNA on a target gene's expression. The data type is novel, and there is a lack of and a need for principled and sound guidelines for how to best analyze these data. Candidate options include (i) competent assembly of known statistical building blocks, (ii) repurposing more specialized tools that are known to work for other data types such as RNA-seq differential expression, and (iii) entirely new statistical methodology.

The ms in its current form seems to claims to be (iii). In fact, it is closer to (i), yet fails to take into account statistical "basics", and ends up reinventing the wheel, which turns out to be a bit eccentric, as explained below.

*We thank the Reviewer for their thorough appraisal and thoughtful comments. While we agree with some of the specific points, outlined below, we believe that we have also not explained the nature of the data nor our methodology clearly enough, and thus, have not provided enough information for the Reviewer. We address this in more detail in the specific points below and in the revised manuscript.*

*We definitely leverage numerous existing statistical tools in our method (e.g., linear optimization of a variable), but do believe that MAUDE represents a non-trivial combination of building blocks designed to models these unique data and provide a superior alternative to previous methods.*

Specific points:

1.) The main claim to novelty of this ms is the statistical method, outlined on page 4 line 54 (4:54) until 6:8 and 13:32-13:49. However, the procedure looks similar to mixture modeling	and	the	EM-algorithm	(e.g.,	Raftery's	work, <https://www.stat.washington.edu/raftery/Research/mbc.html>	and [https://stat.washington.edu/mclust](https://stat.washington.edu/mclust),)), but with less mathematical sophistication and more heuristics. It would be important to put the current work into such context and highlight what is new or different.

*We thank the Reviewer for this point. While MAUDE shares the “optimization” goal with Expectation-Maximization (EM), MAUDE does not use EM and is not even iterative. (Even if it did use EM, many very important bioinformatic tools are based on EM because it has proven to be extremely useful in many applications (e.g., RSEM for RNA-seq analysis as just one example.)*

*In particular, MAUDE has to take a fundamentally different approach than mixture modeling because the underlying data have different properties, and were generated by different processes. In contrast to mixture modeling, we model a set of dependent samples arising from discrete distributions. In particular, data were discretized from cell sorting experiments such that cells with expression levels falling within specific were sorted into discrete bins. They were discretized over only part of the distribution, so there are “blind spots”, where no measurements were taken. The guide abundance in each bin was then quantified by sequencing, adding another layer of complexity, since individual sequencing reads may come from the same source molecule (and so are not independent). We have clarified the nature of the data in the text (p. 4).*

Even more fundamentally, the basic problem that the method is trying to solve is to test for a difference between two empirical distributions, and it is not clear that any of the attempted sophistication is actually needed. There is a plethora of statistical tests (and literature, experience) for this basic question, such as t-test, Wilcoxon test or KS-test. In the authors' comparison, only the heavy tanks edgeR, DESeq2 and a so-called "original" log-ratio method are considered. If the proposed method turns out to be better than these basic statistical tests, it would be helpful to get some interpretation of "why?".

*We thank the Reviewer for this comment. To clarify, our data does not consist of sets of continuous samples coming from each underlying distribution that could be used for these tests. These continuous samples existed in the sorting experiment (determining which sorting bin the cells ended up in), but were lost thereafter, and so must be reconstructed from the data we do have. Instead, our data consists of read count distributions over the sorting bins and input. For example, for guide X, we might see 12 reads in bin A, 6 in B, and 10 in the input. To apply any of the tests suggested, we would have to roll out these values into a single vector (ELA, ... {12 times total} ..., ELA, ELB, ... {6 times total} ..., ELB), where ELA and ELB represent the average expression of bins A and B, and compare that to a similar vector created for some null distribution. The nature of these two vectors violates many of the assumptions of these tests. Specifically, all of the suggested statistical tests are violated by the facts that the data are not independent samples (because one sorted cell could result in many reads), and have ties (because the expression measurements represent discrete bins). As a result, any of these tests would vastly overestimate the significance of everything. Indeed, this is why algorithms for analyses of sequencing data (e.g., RNA-seq) do not use these tests.*

*Further, the above analyses would entirely fail to account for the fact that the abundance within each bin needs to be relative to the total abundance because we can increase the number of reads just by sequencing more deeply within a bin (e.g., maybe we saw twice as many reads for guide X in A because we got twice as many reads total in bin A). Thus, we need to scale the reads in each bin to a relative frequency. At this point, comparing the relative abundance in A to that in B is the log(fold-change) (“original”) approach. However, there was important statistical information present in the actual read numbers; we can be more confident in a ratio of 100:10 reads than a ratio of 10:1. EdgeR and DESeq2 are indeed used to account for the importance of read counts, while still comparing the relative abundances in A and B to find a relative skew.*

*However, while the EdgeR and DEseq2 approaches return an estimated statistical significance for each guide, they cannot be generalized to more sorting bins, and fail to account for the overall abundance of the guides in the experiment, which can greatly impact overall interpretation of the data. Further, these approaches do not provide an estimated expression level for each guide – only the log fold change for the skew between the two bins. It is exactly these problems that MAUDE set out to address.*

*We have clarified these points in the text (p. 3). We also note that we have rephrased the “original” approach to better describe what that approach is (“log(high/low)”; throughout).*

2.) The description of the experimental designs and the data that this method is intended for could be more explicit. It is implicit in the manuscript, but readers would benefit from a clearer delineation, perhaps in lieu of the list of assumptions at the beginning of the Results section.

*We agree with the Reviewer and have made this clearer by adding a paragraph to the beginning of the Results describing the nature of the data (p. 4).*

Indeed, this list of assumptions is troubling since it does not indicate which of these assumptions is sufficient or necessary, and no formal proof is given. From a practical point of view, users would be either interested in necessary assumptions, i.e., assumptions which if not met imply that the method is definitely invalid, or verifiable sufficient assumptions, i.e., assumptions that can be demonstrated to hold. The assumptions that are actually stated are, however, neither of this. They are at the same time too strong (i.e., the method is likely to essentially function even if they do not hold) and unverifiable. I believe, however, that this might be primarily a problem of presentation, and think that this part of the ms could be best fixed by (i) replacing "assumptions" by a description of the experimental designs and the properties of the data that the method is designed for; and (ii) adopting an "ends justify the means" approach, i.e., to show the validity of the method by the validity/quality of its outputs, rather than of its inputs.

*We fully agree with the Reviewer and have followed their excellent suggestions and rephrased this section as described above (p. 4).*

3.) The Stouffer method for combining z-scores is unrobust, e.g., in a situation with 99 small z-scores and 1 that is very large, their sum (Stouffer method) can still be large, and thus "significant"; and this seems unsatisfactory.

*We thank the Reviewer for this very important question.*

*First, we do note that the Stouffer method is not simply a sum of the Z scores; it is their sum divided by the square root of the number of Z scores. Accordingly, with 100 Z scores, all but one of which fall near 0 and the remaining one has Z=10 (p <10-23), their*

*Stouffer Z score would be Z=10/sqrt(100) = 10/10 = 1 (p=0.16). So in that particular example, their Stouffer Z score would not be “significant”.*

*Nevertheless, we appreciate the Reviewer’s critique and recognize that experimental noise can result in outliers. We take three approaches to protect against this:*

*We add a pseudocount to the reads in each bin, acting as a prior favoring guide Z-scores near 0. Accordingly, Z scores can only get extreme in proportion to the amount of support in the underlying data. (p. 6).*

*Stouffer’s method is noisier when combining fewer Z scores. Accordingly, we calibrate element-level Z scores to a matched null distribution using the same numbers of guides per element. For instance, elements with 100 guides are calibrated with a null distribution of Stouffer Z scores, each calculated with 100 random samples of the negative control guides. If the negative control guides include outliers, this will greatly reduce the impact of the same kinds of outliers present in the targeting guides. (p. 7)*

*We recommend comparing experimental replicates and only considering those found to be significant in both. We have made this clear in the text (p. 7).*

4.) Assumption "the perturbations do not differentially affect cell viability": I understand that this is partly because you use the unsorted input library as a reference. Would it be possible to determine the guide distribution in the cell pool just before sorting for use as a reference? The timepoint before cell sorting should in any case be included as a control, even if it is only to show that the assumption is met. Can the authors illustrate this assumption in an experimental dataset?

*We thank the reviewer for pointing this out. Actually, the “input” is usually from a sampling of the same cells used for sorting, and so this assumption is not needed. We have clarified this in the text, and mentioned that the input cells should be taken at the time of sorting (p. 4).*

5.) Assumption "expression is distributed approximately (log) normally". Please show (real) data where this is the case. Both normal and log-normal distributions are implausible (indeed impossible) for count based data, which are non-negative and discrete. A more commonly used and apparently useful model for count data are the Gamma-Poisson or Negative Binomial distributions, why are these not used here?

*We regret the lack of clarity here. We were referring to the expression distribution as measured by flow cytometry. It is not count based, but a readout of fluorescence intensity and often follows an approximate normal distribution. We actually do use a negative binomial distribution to model the count data (p. 6).*

*As Reviewer 2 points out above (and we show in Figure 2), the assumption of lognormal expression is not strictly necessary. Approximate (admittedly, not well defined) log-normality is a common assumption for these kinds of data. Below are examples of approximately log-normal expression distributions from flow cytometry data (taken from the first few Google image search results of “flow cytometry expression distribution”):*

*(*[*https://www.researchgate.net/figure/Cellular-distribution-of-DCIR-A-Flow-cytometry-analysis-of-DCIR-expression-on_fig3_44656033*](https://www.researchgate.net/figure/Cellular-distribution-of-DCIR-A-Flow-cytometry-analysis-of-DCIR-expression-on_fig3_44656033))*)*

([*https://www.researchgate.net/figure/CD150-distribution-on-CLL-B-cells-A-Flow-cytometry-analyses-of-cell-surface-CD150_fig1_320253107*](https://www.researchgate.net/figure/CD150-distribution-on-CLL-B-cells-A-Flow-cytometry-analyses-of-cell-surface-CD150_fig1_320253107))*)*

6.) "we require [...] that the screen includes non-targeting control guides that should not affect expression." In the simulations, the authors use 1000 targeting guides and 1000 non-targeting control gRNAs. This ratio of 1.1 is far away from a typical screening library used in this type of screens (in the CD69 publication by Simeonov et al., ~10,900 targeting gRNAs vs. 2,244 controls). I suggest changing the targeting.control ratio in the simulations to make it more realistic.

*We thank the Reviewer for this point. However, we believe that the relevant parameter is actually the ratio of active to inactive guides, and the overall number of negative control guides. The ratio of active to inactive guides can alter the overall expression distribution of the cells being sorted. The active:inactive ratio in our simulation is actually very similar to the CD69 screen. In the case of our simulation, we have 1,000 negative control guides, and 1,000 targeting guides, 500 of which alter expression (500/2000 or 25% of all guides). In the CD69 data, 2,683 of 12,889 guides were within a region identified by MAUDE as being active (FDR<0.1 in both experimental replicates), comprising 20.8% of all guides. Thus, both the simulated and real data have a similar overall fraction of active guides.*

*The primary use of the negative control guides is to calibrate the statistics. For instance, we take the median expression of the negative control guides to center the expression levels of the individual guides (to get the guide Z score; p. 6). We also use the negative control guides for calibrating the statistical significance of our element Stouffer Z scores by repeatedly sampling the negative control guides to produce “null” Stouffer Z scores*

*for each number of guides per element (p. 7). In both cases, more guides produce more robust results. We aimed to be conservative by using the low end of what is normally used for such screens (1,000 negative control guides). We have elaborated on this design choice in the text (p. 15).*

7.) In their analysis of CD69 regulation, the authors state that their method is able to detect new regulatory elements. However, this example only concerns one gene, there is no experimental validation, and the statistics in Fig. 3A-D are also only calculated on this one example. It would be more convincing if the authors could showcase the benefits of MAUDE in multiple independent datasets.

*We wholeheartedly agree with the Reviewer on this point. However, as exciting as this approach is, the field is still emerging and there are simply not that many published studies. Of the existing studies, none report all the data needed for MAUDE analysis. Some lack negative control guides, and many did not report the sorting bin positions and bin fractions. While these data are readily available to the experimenter, they are not commonly included in the supplementary data. Even in the case of the CD69 data that we analyzed, the bin fractions were not provided; we had to reverse engineer the bin sizes from a supplementary figure (Extended Data Fig. 1a) that displayed the bin positions on a cell density distribution (p. 15). In this same paper, Simeonov et al analyzed a second gene (IL2RA), but did not include the bin sizes or display the bin positions, and so we could not re-analyze that data. We hope that, going forward, bin parameters will become a standard parameter to report.*

*Since the time of our original submission, a study from our own group that is appropriate for MAUDE analysis was concluded (Ray et al, Nature Communications; accepted for publication). Here, we had both CRISPRi and CRISPRa data for one locus (TNFAIP3) done in duplicate, for four cell lines, using Flow-FISH (a method to perform CRISPR screens through sorting cells based on their RNA expression of the target gene; Fulco, et al. Nature Genetics 2019) to read out TNFAIP3 expression. Including these data, we now have ten experiments with different experimental parameters with which to test MAUDE, leading to much greater confidence in our results. Although these new data are significantly noisier than the CD69 screen data (likely attributable to the RNA-based expression readout (Flow-FISH)), they show the same trend: MAUDE outperforms the other methods in >80% of cases. We have described this new analysis on p. 9 and in Figure 3.*

*For the Reviewer’s reference we also provide a list of the existing datasets and the reasons MAUDE analysis could not be applied:*

| *Study* | *PMID* | *Reason MAUDE could not be applied* |
| --- | --- | --- |
| *Konermann 2015* | *25494202* | *Selection-based screen* |
| *Fulco 2016* | *27708057* | *Selection-based screen* |
| *Fulco 2019* | *31784727* | *Missing input guide abundance sequencing* |
| *Sanjana 2016* | *27708104* | *Selection-based screen* |
| *Parnas 2015* | *26189680* | *Missing bin fractions* |
| *Klann 2017* | *28369033* | *No negative control guides* |
| *Canver 2015* | *26375006* | *No raw data or bin fractions provided* |
| *Simeonov 2017* | *28854172* | *Missing bin fractions (IL2RA)  In original submission (CD69)* |
| *Korkmaz 2016* | *26751173* | *Selection-based screen* |
| *Diao 2017* | *28417999* | *Missing bin fractions; partially missing raw data* |

8.) I highly appreciate the fact the authors provide an R package via their GitHub page. The documentation (manual pages, examples, vignette, unit tests) seem minimalistic, and to really become a re-usable tool, that should in my opinion be extended.

*We greatly appreciate the Reviewer reviewing our online documentation. We have expanded the online examples to include more detailed forms of data analysis.*

Minor comment:

Line 45: missing word / typo: Elements can defined a priori,

*Thank you. We have fixed this typo.*

**Second round of review**

**Reviewer 1**

The authors have significantly improved the manuscript in the revision. All my questions have been adequately addressed.

**Reviewer 2**

The presentation of the work has been significantly improved, I now find it much clearer to understand what the authors are trying to achieve.

I have two remaining comments. I do not want to be the person that unreasonably delays publication, but the below two suggestions are good practice in the field of bioinformatics, and are in line with 'open science' principles. This paper is from one of the finest institutions in the country, from one of the most excellent and best-organized labs in that institution, so I think it is not unreasonable to hold them to the same standards as we do with any researcher from obscure places overseas.

1. A central part of this manuscript is the presentation of the software tool MAUDE, an R package that the authors currently distribute via their GitHub page. This is a solution inferior to submitting the package to a public repository like Bioconductor or CRAN, which provide automated quality and functionality checking, a smoother installation process, and proper life cycle and version management. I suggest the authors submit their package to one of these (Bioconductor seems appropriate).

2. Another important part of the manuscript from the point of view of readers is the section "MAUDE outperforms other approaches..." and Fig.3. An Rmarkdown file that reproduces this method comparison and produces the measurements (AUROC etc.) underlying Fig.3 should be provided for reproducibility.

Minor:
- the phrase 'actual data from a two published datasets' (line 9:13) contains a typo, but also sounds tautological. Why not just 'two published datasets'?

**Authors Response**

**Point-by-point responses to the reviewers’ comments:**

**Reviewer 2**

The presentation of the work has been significantly improved, I now find it much clearer to understand what the authors are trying to achieve.

I have two remaining comments. I do not want to be the person that unreasonably delays publication, but the below two suggestions are good practice in the field of bioinformatics, and are in line with 'open science' principles. This paper is from one of the finest institutions in the country, from one of the most excellent and best-organized labs in that institution, so I think it is not unreasonable to hold them to the same standards as we do with any researcher from obscure places overseas.

1. A central part of this manuscript is the presentation of the software tool MAUDE, an R package that the authors currently distribute via their GitHub page. This is a solution inferior to submitting the package to a public repository like Bioconductor or CRAN, which provide automated quality and functionality checking, a smoother installation process, and proper life cycle and version management. I suggest the authors submit their package to one of these (Bioconductor seems appropriate).

*We are also planning to submit MAUDE to bioconductor. We agree with Reviewer 2 that this will help ensure MAUDE has a lasting impact. However, this process is still ongoing. Since this will only change the installation instructions on the MAUDE GitHub page and we have already included several versioning and installation options, we agree with you that this should not be a barrier to publication. If you would like, we would be happy to update you when this process is completed.*

2. Another important part of the manuscript from the point of view of readers is the section "MAUDE outperforms other approaches..." and Fig.3. An Rmarkdown file that reproduces this method comparison and produces the measurements (AUROC etc.) underlying Fig.3 should be provided for reproducibility.

*We have uploaded the Rmarkdown file to the MAUDE github page that recreates the entirety of the method comparison analysis in Fig. 3A-C (https://carldeboer.github.io/MAUDE/Evaluation/method_evaluation.html)*

Minor:
- the phrase 'actual data from a two published datasets' (line 9:13) contains a typo, but also sounds tautological. Why not just 'two published datasets'?

*We have revised the awkward sentence noted by Reviewer 2 (line 9:13).*
